# Conditional cancer-specific mortality in T4, N1, or M1 prostate cancer: implications for long-term prognosis

**DOI:** 10.1186/s13014-015-0470-0

**Published:** 2015-07-30

**Authors:** Vinayak Muralidhar, Brandon A. Mahal, Paul L. Nguyen

**Affiliations:** Harvard-MIT Division of Health Sciences and Technology, Harvard Medical School, Boston, MA 02115 USA; Harvard Medical School, Boston, MA 02115 USA; Department of Radiation Oncology, Dana-Farber Cancer Institute and Brigham and Women’s Hospital, 75 Francis St, Boston, MA 02115 USA

**Keywords:** Prostate cancer, Conditional survival, Racial disparities

## Abstract

**Background:**

The risk of prostate cancer-specific mortality (PCSM) following a diagnosis of prostate cancer may improve after patients have survived a number of years after diagnosis. We sought to determine long-term conditional PCSM for patients with stage T4, N1, or M1 prostate cancer.

**Methods:**

We identified 66,817 patients diagnosed with stage IV (T4N0M0, N1M0, or M1) prostate cancer between 1973 and 2011 using the Surveillance, Epidemiology, and End Results (SEER) database. Conditional five-year PCSM was evaluated for each group of patients at 5, 10, and 15 years of survival according to the Fine & Gray model for competing risks after adjusting for tumor grade, age, income level, and marital status. Race-stratified analyses were also performed.

**Results:**

There were 13,345 patients with T4 disease, 12,450 patients with N1 disease, and 41,022 patients with M1 disease. Median follow-up among survivors in the three groups was 123 months (range: 0–382 months), 61 months (range: 0–410 months), and 30 months (range: 0–370 months), respectively. Conditional PCSM improved in all three groups over time. Among patients with T4 disease, 5-year PCSM improved from 13.9 % at diagnosis to 11.2, 8.1, and 6.5 % conditioned on 5, 10, or 15 years of survival, respectively (*p* < 0.001 in all cases). In patients with N1 disease, 5-year PCSM increased within the first five years and decreased thereafter, from 18.9 % at diagnosis to 21.4 % (*p* < 0.001), 17.6 % (*p* = 0.055), and 13.8 % (*p* < 0.001), respectively. In patients with metastatic disease, 5-year PCSM improved from 57.2 % at diagnosis to 41.1, 28.8, and 20.8 %, respectively (*p* < 0.001). White race was associated with a greater increase in conditional survival compared to non-white race among those with T4 or N1 disease.

**Conclusions:**

While patients with T4, N1, or M1 prostate cancer are never “cured,” their odds of cancer-specific survival increase substantially after they have survived for 5 or more years. Physicians who take care of patients with prostate cancer can use this data to guide follow-up decisions and to counsel newly diagnosed patients and survivors regarding their long-term prognosis.

## Introduction

Stage IV prostate cancer, consisting of stage T4 (invasion of adjacent organs), N1 (regional nodal spread), or M1 (metastatic spread) disease [1], is a relatively rare diagnosis, accounting for approximately 5 % of prostate cancer diagnoses [2]. When a patient is diagnosed with such unfavorable cancer, he may appropriately ask about his prognosis, which depends on many factors including his specific tumor status, ability to tolerate cancer therapy, and his competing health risks. One complicating factor is that most cancer prognostic information is reported from the time of diagnosis and may overestimate the risk of mortality for the patient who has already survived their disease for some time [3]. It can therefore be challenging, but important, to determine a patient’s changing prognosis as he lives years past his diagnosis. Compounding this challenge in patients with T4, N1, or M1 prostate cancer is that due to their relative rarity, some clinicians may have less clinical experience to rely on when counseling patients about their long-term prognosis.

Some data suggests that patients with T4, N1, or M1 disease are unique in the degree to which prognosis improves as patients survive their disease [4]. It is likely that in each of these heterogeneous groups, patients with worse disease die more quickly, such that the cancer-specific survival for the remainder of the patients is relatively better and therefore captured by the conditional survival at 5–15 years following diagnosis. Most studies on conditional mortality after a prostate cancer diagnosis to-date have focused on either localized or distant disease, with no studies to our knowledge specifically examining the conditional mortality of patients with T4 or N1 disease [5–8]. In addition, studies have usually reported mortality conditioned on 5 years or occasionally 10 years of survival, but not as long as 15 years [9]. In this study, we determine the prognosis of prostate cancer patients with T4, N1, or M1 disease conditioned on up to 15 years of survival. As a secondary aim, we also determine the interaction between race and conditional mortality to study the possibility that barriers to long-term cancer follow-up among minorities might affect how long-term prognosis changes over time [10].

## Methods

### Patient population

The Surveillance, Epidemiology, and End Results (SEER) database collects cancer diagnostic, treatment, and survival data from 18 SEER registries, accounting for approximately 28.0 % of the US population [11]. We used the SEER*Stat 8.1.5 software to extract cases from the SEER database. This study was approved by the Institutional Review Board at the Dana-Farber Cancer Institute.

Patients were included if they were diagnosed with stage T4N0M0, N1M0, or M1 prostate cancer between 1973 and 2011. In total, this approach identified 66,817 men. We collected information from SEER on tumor characteristics, including clinical staging information and grade. We also extracted patient characteristics, including survival information, age at diagnosis, marital status at diagnosis, race, median family income in the county of diagnosis, cause of death, and length of survival.

### Statistical analysis

Stata/MP 13.1 was used for all statistical analyses. Patient demographic data were summarized for patients with T4, N1, or M1 disease. Prostate cancer-specific mortality was estimated using the Fine & Gray model [12] for competing risks, adjusting for marital status, race, age, median family income in the county of diagnosis, and tumor grade. Conditional mortality at time *T* was determined by identifying patients who survived for *S > T* months and generating a new survival variable *S’ = S - T.* Survival analysis was then performed on patients who survived past time *S* using *S’* as the survival variable and compared to the general cohort using *S* as the survival variable. This procedure was repeated for *T* = 5, 10, or 15 years. We also analyzed subgroups of patients with M1 disease based on whether they had M1a disease (distant lymph node involvement), M1b disease (bone involvement), or M1c disease (other site involvement with or without bone involvement) [1]. Analyses were also repeated following stratification by race (white versus non-white). Interaction analysis was performed as has previously been reported [13], namely by modeling the effect of a new variable race*SurvivalT, where *SurvivalT* represents whether or not a patient survived past time *T*. P-values were reported as statistically significant if less than α = 0.05 after correction for multiple comparisons, when applicable [14]. Specifically, we used α = 0.05/3 = 0.0167 for the calculations of 5-year PCSM conditioned on 5, 10, or 15 years of survival.

## Results

### Patient characteristics

There were 13,345 patients with T4 disease, 12,450 patients with N1 disease, and 41,022 patients with M1 disease. Median follow-up among survivors in the three groups was 123 months (range: 0–382 months), 61 months (range: 0–410 months), and 30 months (range: 0–370 months), respectively. Other baseline characteristics of the three cohorts and of those who survived for 5, 10, or 15 years are represented in Table 1.

**Table 1:** Characteristics among patients with T4, N1, or M1 disease at diagnosis and at 5, 10, and 15 years of survival

T4				
	At diagnosis	5 years	10 years	15 years
N	13,345	7,921	4,839	2,743
% White	81.1 %	82.1 %	83.7 %	86.3 %
Median age at diagnosis	68	67	66	65
% Married	76.8 %	81.7 %	84.1 %	86.6 %
Median family income in county of residence	$53,880	$54,020	$54,470	$55,470
% Low-grade (Gleason score less than 7)	51.0 %	65.4 %	73.9 %	78.1 %
N1				
	At diagnosis	5 years	10 years	15 years
N	12,450	6,791	3,069	1,196
% White	83.4 %	84.1 %	85.2 %	86.2 %
Median age at diagnosis	65	65	65	64
% Married	78.5 %	81.5 %	84.5 %	87.0 %
Median family income in county of residence	$53,440	$53,880	$54,470	$56,490
% Low-grade (Gleason score less than 7)	33.3 %	46.1 %	57.4 %	62.0 %
M1				
	At diagnosis	5 years	10 years	15 years
N	41,022	7,514	1,960	525
% White	75.6 %	75.2 %	74.0 %	77.3 %
Median age at diagnosis	72	70	69	67
% Married	65.5 %	73.3 %	77.9 %	83.2 %
Median family income in county of residence	$53,440	$54,020	$56,960	$58,140
% Low-grade (Gleason score less than 7)	28.7 %	46.8 %	61.1 %	66.5 %

### Conditional mortality improves over time among patients with T4, N1, or M1 prostate cancer

**Fig. 1:**
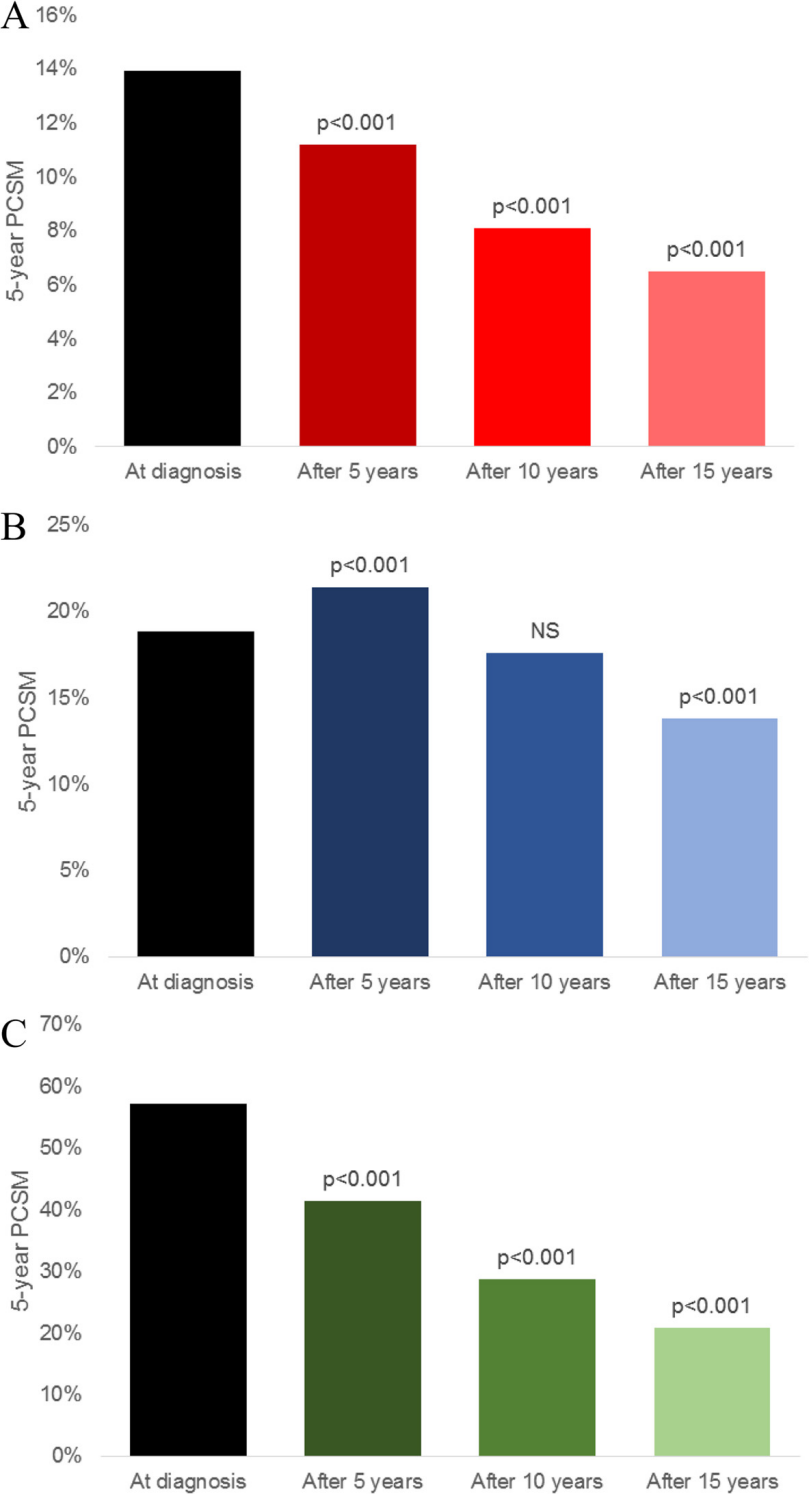
Five-year PCSM among patients with T4 (**a**), N1 (**b**), or M1 (**c**) prostate cancer at diagnosis and conditioned on 5, 10, or 15 years of survival

Conditional adjusted prostate cancer-specific mortality improved among patients with T4, N1, or M1 disease over time (Table 2 and Fig. 1). Among patients with T4 disease, 5-year PCSM improved from 13.9 % at diagnosis to 11.2, 8.1, and 6.5 % conditioned on 5, 10, or 15 years of survival, respectively (*p* < 0.001 in all cases). In patients with N1 disease, 5-year PCSM slightly worsened within the first five years, from 18.9 % at diagnosis to 21.4 % conditioned on 5 years of survival (*p* < 0.001), but then declined to 17.6 % (*p* = 0.055) and 13.8 % (*p* < 0.001) conditioned on 10 and 15 years of survival, respectively.

**Table 2:** Five-year adjusted conditional PCSM for patients with T4, N1, or M1 disease

5-year PCSM	T4	N1	M1
at diagnosis	13.9 % (ref)	18.9 % (ref)	57.2 % (ref)
after 5 years	11.2 % (*p* < 0.001)	21.4 % (*p* < 0.001)	41.4 % (*p* < 0.001)
after 10 years	8.1 % (*p* < 0.001)	17.6 % (*p* = 0.055)	28.8 % (*p* < 0.001)
after 15 years	6.5 % (*p* < 0.001)	13.8 % (*p* < 0.001)	20.8 % (*p* < 0.001)

**Fig. 2:**
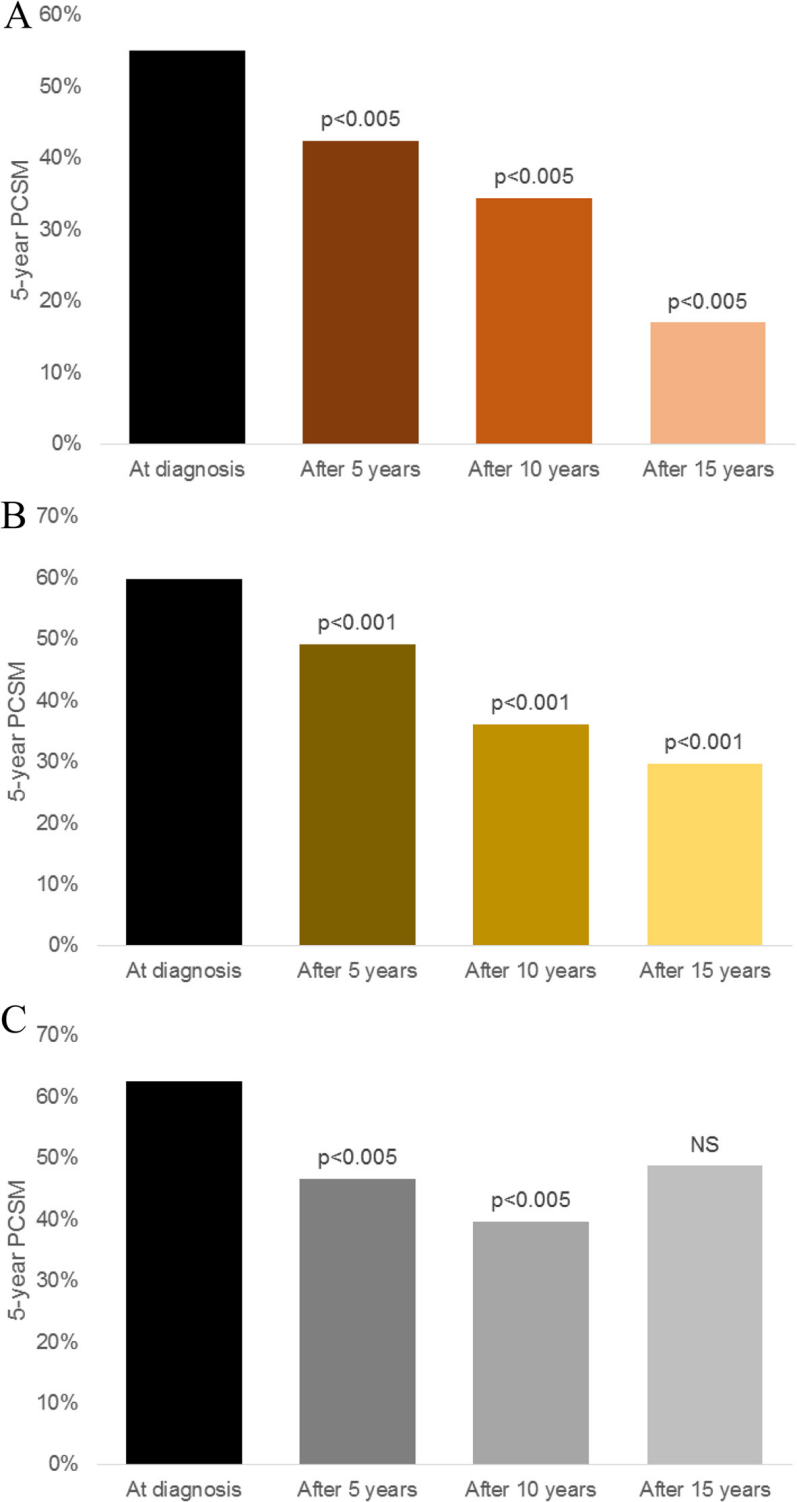
Five-year PCSM among patients with M1a (**a**), M1b (**b**), or M2c (**c**) prostate cancer at diagnosis and conditioned on 5, 10, or 15 years of survival

In patients with metastatic disease, 5-year PCSM improved from 57.2 % at diagnosis to 41.1, 28.8, and 20.8 % conditioned on 5, 10, and 15 years of survival, respectively (*p* < 0.001 in all cases). In subgroup analyses (see Table 3 and Fig. 2), patients with M1a disease (*N* = 3,950) had improved 5-year PCSM from 55.0 % at diagnosis to 42.4, 34.4, and 17.0 % at 5, 10, and 15 years of survival, respectively (*p* < 0.005 in all cases). For the subgroup of patients with M1b disease (*N* = 14,413), 5-year PCSM improved from 59.7 % at diagnosis to 49.1, 36.0, and 29.7 % at 5, 10, and 15 years of survival (*p* < 0.001 in all cases). For the subgroup of patients with M1c disease (*N* = 4,130), 5-year PCSM improved from 62.6 % at diagnosis to 46.5 % and 39.6 % at 5 and 10 years of survival, respectively (*p* < 0.005 in both cases), with no significant difference at 15 years of survival (48.7 %, *p* = 0.221).

**Table 3:** Five-year adjusted conditional PCSM for patients with M1a, M1b or M1c disease

5-year PCSM	M1a	M1b	M1c
at diagnosis	55.0 % (ref)	59.7 % (ref)	62.6 % (ref)
after 5 years	42.4 % (*p* < 0.005)	49.1 % (*p* < 0.001)	46.5 % (*p* < 0.005)
after 10 years	34.4 % (*p* < 0.005)	36.0 % (*p* = 0.055)	39.6 % (*p* < 0.005)
after 15 years	17.0 % (*p* < 0.005)	29.7 % (*p* < 0.001)	48.7 % (*p* = 0.221)

### White patients derive a larger conditional mortality benefit than non-white patients

In order to determine whether race interacted with survival, we repeated our initial analyses after stratifying by race and by modeling an interaction term. Among patients with M1 disease, conditional mortality was similar between white and non-white patients (data not shown). However, among patients with N1 disease, non-white patients did not have a significant reduction in conditional mortality at 5, 10, or 15 years of survival compared to diagnosis (adjusted HR = 1.16 [*p* = 0.051], AHR = 1.04 [*p* = 0.686], and AHR = 0.881 [*p* = 0.593], respectively), while white patients had similar mortality as the overall cohort, with adjusted hazard ratios of 1.10 (*p* < 0.001), 0.898 (*p* = 0.022), and 0.712 (*p* < 0.001), respectively. While non-white patients with T4 disease had improved 5-year PCSM after having survived 5, 10, or 15 years, their improvements were 20-39 % smaller than those of white patients (*p* < 0.05 by interaction analysis).

## Discussion

Among patients with T4, N1, or M1 prostate cancer, we found that 5-year prostate cancer-specific mortality (PCSM) generally improved among all three groups conditioned on 5, 10, or 15 years of survival (with the exception of patients with N1 disease conditioned on 5 years of survival), even after adjusting for patient-specific factors like tumor grade, age, marital status, county-wide median income, and race. Interestingly, we found that this relationship was stronger in white patients than among non-white patients with T4 or N1 disease.

These data may be of great interest to physicians and patients. Our survival analyses imply that cancer-specific mortality is not static after diagnosis with advanced prostate cancer. While it may not be appropriate to label patients with advanced-stage prostate cancer as “cured” after surviving for several years, many of these patients are at significantly reduced likelihood of cancer-specific death the longer they survive. Physicians should use results to better counsel patients and provide appropriate follow-up and surveillance. For example, we found that cancer-specific mortality drops sharply among patients with T4 disease after 5, 10, and 15 years of survival. Therefore, it would be appropriate for clinicians to increase the time between follow-up appointments, imaging, and laboratory tests once patients survive to 5 years. On the other hand, we found that patients with N1 disease did not have a reduction in PCSM until 10 years. Therefore, physicians should monitor the N1 patient who has survived for 5 years just as closely as they would monitor the newly diagnosed one.

Patients can also use this data to better understand how their long-term prognosis changes over time. Most cancer-specific mortality data is reported from diagnosis. Therefore, it can be difficult to re-assess prognosis for the fortunate patient who is able to survive 5, 10, or even 15 years after diagnosis. Our data can help patients understand their changing risk of PCSM as time passes. For example, a patient newly diagnosed with metastatic prostate cancer would likely be counseled that their risk of dying within five years is 50 % or more [15]. In contrast, based on our data, one who has survived for 5 years already should be counseled that their risk of dying in the next five years is much lower. Counseling patients about their improving risk of mortality over time may have important psychological and emotional benefits for patients and their families [16, 17].

In addition to our findings that conditional survival improved among all three groups of patients, our secondary analyses may point to racial disparities in long-term prostate cancer care. We found that among patients with T4 disease, non-white patients had smaller improvements in survival than white patients; among those with N1 disease, non-white patients had no improvement at all. While others have shown that minorities are at higher risk of cancer-specific mortality [18], our results show that the differences may grow proportionally larger as time goes on. These differences may be due to racial disparities in follow-up surveillance following initial cancer treatment and differences in the eventual receipt of salvage therapy. Minorities have previously been shown to report more barriers to follow-up care after completing cancer treatment [10]. Our data suggest that these differences in follow-up may translate to smaller reductions in cancer-specific mortality over time, resulting in relatively more cancer-specific death among non-white patients compared to white patients. Physicians should therefore pay special attention to minority patients with advanced prostate cancer to make sure they receive excellent long-term follow-up and surveillance in order to help reverse this cause of increased cancer-specific mortality.

To our knowledge, other studies examining conditional survival from prostate cancer have not specifically examined patients with T4 or N1 disease, two important subsets of patients with whom some clinicians may have limited experience due to their relative rarity [4–7, 9]. Some authors have reported the conditional outcomes of all stage IV patients together [4, 8], but because the subsets of stage IV patients have very heterogeneous outcomes [15] the data presented here may be more clinically applicable since we have separately considered T4, N1, and M1 disease. One study in patients with pathologic N1 disease demonstrated that conditional freedom from biochemical recurrence rapidly improves within the first five years after diagnosis but did not report mortality data [19]. In addition, we calculated 5-year PCSM conditioned on up to 15 years of survival, whereas most previous studies have stopped at 5 years of survival. Our results for the subset of patients with M1 disease are consistent with the results of similar studies [6, 8, 9], with some variation in the estimates of PCSM referable to differences in cohort selection (e.g. country of study) and statistical techniques (e.g. use of Fine & Gray’s model with adjustment for patient demographic factors in our study versus calculation of excess mortality or use of life tables in other studies). Our subgroup analyses of patients with M1a, M1b, or M1c disease showed moderate heterogeneity in the conditional mortality estimates. These results are consistent with the work others showing somewhat differing survival outcomes among these three groups [20, 21], with M1c conferring the worst prognosis overall and the smallest improvements in conditional mortality out of the three subgroups.

Despite our sample size, our study has some limitations. First, the SEER database does not contain data about disease recurrence or receipt of salvage therapy. Therefore, we were not able to analyze these important secondary outcomes. Second, the SEER database has previously been reported to have coding errors in cancer stage [22], so we may have erroneously included or excluded some patients in our study. While this may reduce the reliability of our results, coding errors are likely to be random and therefore not likely to systematically bias our findings. Third, our cohort includes patients over a very long time period. Improvements in cancer care and disease surveillance (e.g. more sensitive prostate-specific antigen and improved imaging) over the past decades could lead to our data overestimating the PCSM compared to a modern patient’s risk.

## Conclusion

In order to better understand how the cancer-specific mortality risk of advanced prostate cancer changes over time, we studied the conditional mortality of patients with stage T4, N1, or M1 prostate cancer. We found that in all three groups (T4, N1, and M1), cancer-specific mortality generally improved after survival to 5, 10, or 15 years. Among those with T4 or N1 disease, non-white patients had smaller improvements compared to white patients or no improvement at all. Our results should be used to counsel prostate cancer survivors on their changing risk profile and to tailor follow-up and cancer surveillance over time. In addition, physicians should pay special attention to minority patients, as they may be at risk for relatively poor conditional mortality, possibly due to worse long-term follow-up.

## Abbreviations

SEER: Surveillance, Epidemiology, and End Results
PCSM: Prostate cancer-specific mortality
